# Differences in the Urinary Microbiome of Patients with Overactive Bladder Syndrome with and without Detrusor Overactivity on Urodynamic Measurements

**DOI:** 10.3390/life13051199

**Published:** 2023-05-17

**Authors:** Aida Javan Balegh Marand, Cléo Baars, John Heesakkers, Ellen van den Munckhof, Morteza Ghojazadeh, Mohammad Sajjad Rahnama’i, Dick Janssen

**Affiliations:** 1 Department of Urology, Maastricht University Medical Centre (MUMC+), 6229 HX Maastricht, The Netherlands; 2 Department of Urology, Radboud University, 6525 GA Nijmegen, The Netherlands; 3 Research Center for Evidence-Based Medicine, Faculty of Medicine, Tabriz University of Medical Sciences, Tabriz 5165665811, Iran; 4 Viroclinics-DDL Diagnostic Laboratory, 2288 ER Rijswijk, The Netherlands; 5 Department of Urology, St. Elisabeth-Tweesteden Hospital, 5022 GC Tilburg, The Netherlands; sajjad_r@yahoo.com

**Keywords:** microbiome, overactive bladder syndrome, detrusor, urodynamics, incontinence

## Abstract

Introduction: It has been hypothesized that the urinary microbiome might play an important role in OAB. Studies have been conducted on the association between OAB symptoms and the microbiome, although a possible causality still has to be determined. Material and Methods: In this study, 12 female patients, ≥18 years of age, with ‘OAB DO+’ and 9 female patients with ‘OAB DO-’ were included. Patients were excluded if they met one of the following exclusion criteria: bladder tumors and previous bladder operations; sacral neuromodulation; injection of Botox in the bladder; and TOT or TVT operations. Urine samples were collected and stored with patient informed consent and with the approval of the Hospital Ethical Review Board (Arnhem–Nijmegen). All OAB patients underwent urodynamics before collecting urine samples, and the diagnosis of detrusor overactivity was confirmed by two individual urologists. In addition, samples from 12 healthy controls who did not undergo urodynamic evaluation were analyzed. The 16S rRNA V1–V2 region amplification and gel electrophoresis were used to determine the microbiota. Results: 12 of the OAB patients had DO shown on their urodynamic studies; the remaining 9 patients had a normoactive detrusor on their urodynamic measurements. Overall, there were no substantial differences among the demographic characteristics of the subjects. The samples were classified as the following: 180 phyla, 180 classes, 179 orders, 178 families, 175 genera, and 138 species. The least commonly observed phyla were Proteobacteria, with an average presence of 10%, followed by Bacteroidetes with 15%, Actinobacteria with 16%, and Firmicutes with 41%. Most of the sequences could be classified according to the genus level for each sample. Discussion: Significant differences were observed in the urinary microbiome of patients with overactive bladder syndrome who have detrusor overactivity on urodynamics compared to OAB patients without detrusor overactivity and matched controls. OAB patients with detrusor overactivity have a significantly less diverse microbiome and show a higher proportion of *Lactobacillus*, particularly *Lactobacillus iners*. The results imply that the urinary microbiome could be involved in the pathogenesis of a specific phenotype of OAB. The urinary microbiome could be a new starting point to study the causes and treatments of OAB.

## 1. Introduction

Overactive bladder (OAB) is a common diagnosis, affecting up to 12% of women [1]. OAB is a symptom-based diagnosis defined as ‘urinary urgency, with or without urgency urinary incontinence, usually with increased daytime frequency and nocturia, if there is no proven infection or other obvious pathology [2,3]. Traditionally, OAB was often assumed to be synonymous with detrusor overactivity (DO); however, DO is only detected in 50% of females with OAB [4]. This suggests that there are more factors contributing to the development of OAB and that patients with DO form a subgroup of OAB patients. DO might be considered a more severe form on the OAB spectrum [5]. Currently, urinary urgency is considered the cardinal symptom of OAB.

The pathophysiology of OAB has not been fully clarified, whatever it is, but it is considered to be multifactorial with evidence that metabolic syndrome, affective disorder, sex hormone deficiency, gastrointestinal functional disorders and subclinical autonomic nervous system dysfunction may play a role [4]. Moreover, metabolic syndrome has been suggested to contribute to the underlying mechanisms for developing OAB and might be associated with a subtype of OAB [6]. For more than a decade, research has demonstrated that urine is in fact not sterile and that the bladder contains a microbiome, which is not detectable under standard culture conditions [7,8]. In recent years, the presence of a microbiome in the bladder of healthy individuals lacking any symptoms has garnered much attention [9,10]. This is due to findings pointing to how these complex and dynamic microbial ecosystems can be of assistance in maintaining individual health and how a disturbance in these ecosystems may contribute to Lower Urinary Tract Symptoms (LUTS) [7,11]. 

It has been hypothesized that the urinary microbiome might play an important role in OAB [4,11]. Studies have been done on the association between OAB symptoms and the microbiome, although a possible causality still has to be determined. A loss in biodiversity and/or differences in dominant species in female patients with OAB compared to healthy women have been reported [8,11,12,13].

A decrease in *Lactobacillus* was found in five studies [7,8,13,14]. There are different strains of *Lactobacillus*, and *L. gasseri* has been associated with urgency incontinence, while *L. crispatus* may have a more protective effect [7]. In another study in patients suffering from OAB, a decrease in *Streptococcus* compared to healthy subjects was observed [15], and two studies reported increases in Proteus in OAB compared to controls [16].

OAB is a heterogenous symptom complex, and differences in the microbiota between patients with OAB with DO (OAB DO+) and patients with OAB without DO (OAB DO-) have not yet been studied.

The aim of this study is to clarify the differences between the urinary microbiota of these distinct patient groups (OAB DO+ and OAB DO-) and compare them with healthy controls.

## 2. Material and Methods

Female patients, ≥18 years of age, with ‘OAB DO+’ (mean age 57 years, range 31–72) and ‘OAB DO-’ (mean age 56 years, range 24–74) were included in our study. Patients were excluded if they met one of the following exclusion criteria: bladder tumors and previous bladder operations; sacral neuromodulation; injection of Botox in the bladder; TOT or TVT operations. Urine samples were collected and stored with patient informed consent and with approval of the Hospital Ethical Review Board (Arnhem–Nijmegen). All OAB patients underwent urodynamics before collecting urine samples, and the diagnosis detrusor overactivity was confirmed by two individual urologists. Healthy controls did not undergo a urodynamic evaluation. 

Midstream urine of 21 female patients with OAB, 12 patients with OAB DO+ and 9 patients with OAB DO- were collected. Furthermore, we obtained 12 samples from healthy, age- and sex-matched controls (mean age 53 years, range 38–61) through the biobank of the Nijmegen University Hospital in the Netherlands.

The samples were stored at −80 °C. These frozen sample were shipped in batches on dry ice to the Diagnostic Laboratory (DDL) in Rijswijk, Netherlands, for 16S rRNA gene sequence analysis.

### 2.1. Nucleic Acid Extraction from Urine Samples

DNA was extracted from 500 μL urine using the MagNA Pure 96 DNA and Viral NA Large Volume Kit and the Pathogen Universal Protocol using an elution volume of 50 μL. 50 μL 10× PBS was added to each 500 μL sample prior to the extraction to improve DNA extraction efficiency. The DNA was 10 times diluted for the PCR reaction.

### 2.2. 16S rRNA V1–V2 Region Amplification and Gel Electrophoresis

A fragment of ~421 bp of the V1–V2 region of the 16S rRNA gene was amplified using the primers described by Ravel et al. (2010) [17] and Walker et al. (2015) [15] with Illumina overhang adaptor sequences added. Each 50 µL PCR reaction contained 5 µL (10×) Expand High-Fidelity Buffer with 15 mM MgCl_2_ (Roche, France), 2.6 U Expand High-Fidelity Enzyme Mix (Roche), 0.2 mM of each dNTP (Roche), various primer concentrations, and 10 µL of extracted DNA.

The PCR was run for 2 min at 94 °C followed by 35 cycles of 94 °C for 15 s, 55 °C for 30 s, 72 °C for 1 min, and a final extension step at 72 °C for 7 min. The PCR products with a visible band of ~421 bp on gel were subsequently purified and quantified using AMPure XP Beads (Agencourt Bioscience Corporation, Beverly, CA, USA) and the Quant-iT PicoGreen dsDNA Assay Kit (Invitrogen, Paisley, UK), respectively. After library preparation using the Nextera XT kits (Illumina, San Diego, CA, USA), sequencing was performed with the MiSeq desktop sequencer using the MiSeq Reagent Kits v2500-cycles (Illumina). Sequencing data was processed following the QIIME pipeline. Open reference operational taxonomic unit clustering of high-quality sequences (≥100 bp in length with a quality score ≥ Q20) was conducted at a 97% similarity level against a pre-clustered version of the Augustus 2013 Green Genes database. No low-abundance filtering was used.

### 2.3. Statistical Analysis

The relative portion of the different bacteria in the microbiome was calculated for each sample. The microbiome proportions were analyzed to 3 decimal places; a bacterial genus was considered ‘present’ if the proportion was >0.001%.

The Shannon Diversity Index was used as a mathematical tool for calculating the proportional abundance of species in each sample. Quantitative variables are presented as median (interquartile range) values. Qualitative variables are expressed as frequencies or percentages. The normal distribution of the data was assessed using the Kolmogorov–Smirnov and Shapiro–Wilk tests. Kolmogorov–Smirnov and Shapiro–Wilk tests are statistical non-parametric tests that examine the normality of data. A One-Way ANOVA test and a further Tukey analysis were performed to determine the statistical significance of differences in the Shannon numeric between groups [18].

The bacteria were analyzed at genus level, and only *Lactobacillus* was further specified at species level. To evaluate differences in levels of specific bacteria (genus level) between groups, an independent sample Kruskal-Wallis test and further pairwise comparisons of groups were performed. *p* values less than 0.05 were considered to indicate statistical significance. All data were analyzed via SPSS v. 26 software.

## 3. Result

Twelve of the OAB patients had DO diagnosed on their urodynamic studies; the remaining 9 patients had a normoactive detrusor on their urodynamic measurements. Overall, there were no substantial differences among the demographic characteristics of the subjects. The samples were classified as the following: 180 phyla, 180 classes, 179 orders, 178 families, 175 genera, and 138 species.

The least commonly observed phyla were *Proteobacteria*, with an average presence of 10%, followed by *Bacteroidetes* with 15%, *Actinobacteria* with 16%, and *Firmicutes* with 41%. Most of the sequences could be classified to the genus level for each sample.

**Figure 1:**
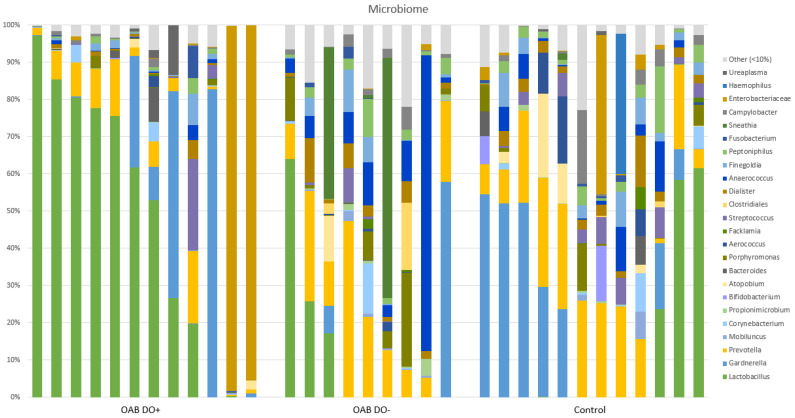
Relative proportions of bacteria in the microbiome from the individual samples. Shows less diversity in the OAB DO+ group and three families in the OAB and control groups; *Lactobacillus*, *Gardnerella,* and *Prevotella*, which were most abundant in the urine samples. *Lactobacillus* was the dominant bacteria of the samples from the OAB DO+ group compared to OAB DO- and control group. *Gardnerella* was the second most abundant. *Prevotella* was most dominant in the control group.

The relative portion of the different bacteria from each sample are summarized in a bar chart (Figure 1). In this bar chart, species which made up less than 10% (meaning the sum of the relative portion of that bacteria from all the samples was less than 10%) are grouped under ‘other’. Species that could not be classified to a genus level were also grouped under ‘other’, except for *Entrobacteriaceae*.

Figure 1 shows that in the OAB DO+ group, 11 of the 12 samples (92%) are dominated by one genus with a relative proportion of at least 50%. In the OAB DO- group and the control group, this is 4 out of 9 (44%) and 5 out of 12 (42%), respectively.

Furthermore, we observed three families in the OAB and control groups; *Lactobacillus, Gardnerella,* and *Prevotella*, which were overall most abundant in the 33 urine samples. More specifically, *Lactobacillus* was observed to be the most dominant bacteria, followed by Gardnerella and then *Prevotella*. *Lactobacillus* was the dominant bacteria (with a proportion of >50%) in 58% of the samples from the OAB DO+ group compared to 11% in OAB DO- group, and 16% in the control group (Figure 1). *Gardnerella* was the second most abundant. Overall, 76% samples showed presence of *Gardnerella*, 83% in the OAB DO+ group, 67% in the OAB DO- group, and 75% in the control group. It should be pointed out that *Prevotella* was most dominant in the control group with a median proportion of 23.5%. In the OAB DO+ and the OAB DO- groups this was 5.1% and 12.6%, respectively (Table 1).

**Figure 2:**
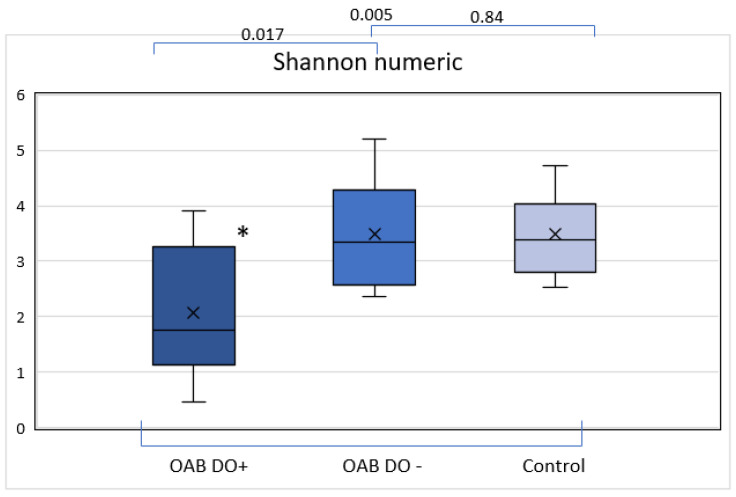
Shannon numeric: OAB DO+, OAB DO-, and control. Showing a statistically significant difference between the Shannon numeric of the OAB DO+ and the other two groups, OAB DO- and control. * Indicating statistically significant difference (*p* < 0.01).

Analysis using the Shannon Diversity Index to evaluate relative microbiome diversity showed an overall median of the Shannon numeric of 3.21. The median Shannon numeric of the OAB DO+, OAB DO-, and control groups were 1.75, 3.34, and 3.39 respectively, as shown in Figure 2. The Kolmogorov–Smirnov and the Shapiro–Wilk test showed that the Shannon numeric was normally distributed in the subgroups. The One-Way ANOVA test showed that the Shannon numeric of the subgroups differed significantly, a further post hoc analysis with a Tukey test showed a statistically significant difference between the Shannon numeric of the OAB DO+ and control groups (*p* = 0.003) and a significant difference between the OAB DO+ and OAB DO- groups (*p* = 0.006). There was no significant difference between the Shannon numeric from the OAB DO- and control group (*p* = 1.00).

The Kolmogorov–Smirnov and Shapiro–Wilk tests showed that the proportions of the different bacteria were not normally distributed. Independent samples of the Kurskal–Wallis tests were used to find significant differences in proportions of bacterial genera between the groups (Table 1). The following genera of bacteria were further analyzed: *Lactobacillus (Iners, Grasseri, and Crispatus*), *Gardnerella, Prevotella, Propionimicrobium, Clostridialis, Dialister, Anaerococcus, Peptoniphilus,* and the family *Enterobacteriacae*.

### 3.1. Lactobacillus

Overall, 16 samples (48.4%) showed the presence of *Lactobacillus* (range 0.005–95.25%), 83.3% in the OAB DO+, 33.3% in the OAB DO-, and 25.0% in the control group. The median proportion of the microbiome formed by *Lactobacillus* in the OAB DO+, OAB DO- and the control group was 57.3%, 0.04% and 0.03%, respectively.

There was a significant difference between the OAB DO+ group and the other two groups (OAB DO- and control, *p* = 0.038 and *p* = 0.011 respectively), with the samples in the OAB DO+ group containing significantly larger proportions.

The *Lactobacillus* was further specified to the species level. The dominant *Lactobacillus* found were *Lactobacillus iners, grasseri*, and *crispatus* in 30%, 15% and 3% of all samples, respectively.

In the OAB DO+ group, 58% showed a dominance of *Lactobacillus iners,* this was 22% and 8% in the OAB DO- and control group, respectively.

### 3.2. Prevotella

*Prevotella* was most dominant in the control group with a median proportion of 23.48% (Table 1). In the OAB DO+ and OAB DO- groups this was 5.13% and 12.58%, respectively. The differences between the OAB DO+ group and the other two groups (OAB DO- and control) were statistically significant (*p* = 0.013 and *p* = 0.004, respectively), with significantly smaller proportions in the OAB DO+ group.

### 3.3. Gardnerella

Overall, 25 samples (75.75%) showed presence of *Gardnerella;* 10 (83%) in the OAB DO+ group, 67 (67%) in the OAB DO- group, and 9 (75%) in the control group. The median proportion of the microbiome formed by *Gardnerella* in the OAB DO+, OAB DO-, and control group was 0.275%, 0.007% and 12.938%, respectively. Statistical testing showed no significant differences between groups for the proportion of *Gardnerella.*

### 3.4. Propionimicrobium

*Propionimicrobium* formed a very small proportion in all groups. Only 4 patients had a proportion of *Propionimicrobium* of more than 1%. Nonetheless, three of those patients were in the OAB DO- group and the difference in proportion between OAB DO+ and OAB DO- was statistically significant (*p* = 0.002) with larger proportions in the OAB DO- group.

### 3.5. Clostridialis

Only 11 samples contained *Clostridialis* and in most, the proportion was less than 1%. Six of these samples were in the OAB DO- group resulting in a median proportion of 0.053%, compared to 0.00% in the other two groups. The difference between the proportions of *Clostridialis* in the OAB DO- and the other two groups (OAB DO+ and control) was statistically significant (*p* = 0.028 and *p* = 0.018).

### 3.6. Dialister

*Dialister* was present in almost all samples, 31 samples in total. However, it was mostly present is small proportions. The median for the OAB DO+, OAB DO- and control group was 0.319%, 2.008%, 2.703%, respectively. Comparison showed a statistically significant difference between OAB DO+ and the other two groups (OAB DO- and control, respectively *p* = 0.003 and *p* = <0.001) with a significantly smaller proportion found in OAB DO+.

### 3.7. Anaerococcus

*Anaerococcus* was present in most samples, 29 in total. The median proportion of *Anaerococcus* in the OAB DO+, OAB DO- and control group were 0.143%, 6.022% and 1.436%, respectively. The proportion in the OAB DO+ group was significantly smaller than in the other two groups.

### 3.8. Peptoniphilus

*Peptoniphilus* was present in all but two samples, mostly in proportions below 5%. However, the proportions were significantly smaller in the OAB DO+ group than in the other two groups with median proportions of 0.274% compared to 2.731% and 2.838% in OAB DO- and control groups, respectively (*p* = 0.011 and *p* = 0.004).

### 3.9. Enterobacteriaceae

*Enterobacteriaceae* was less common, present in 7 of 12 samples (58%) in OAB DO+, 4 out of 9 (44%) samples in OAB DO- and 11 out of 12 samples (92%) in the control group. Only three samples had a *Enterobacteriaceae* proportion of ≥5%, in the OAB DO+ group 2 samples had a proportion of 98.0% and 95.3%, in the control group one sample had a proportion 42.8%. The median proportion of *Enterobacteriaceae* was 0.061%, 0.000% and 0.227% in the OAB DO+, OAB DO- and control group, respectively. The difference between the groups was not statistically significant.

## 4. Discussion

There is a microbiome associated with the healthy urinary tract. This microbiome is thought to differ from pathological states [18]. There is much heterogeneity in literature on microbiome differences in OAB, but there are commonly reported bacterial species reported to be up- and downregulated. Results from this study show a significant reduction in microbiome diversity in OAB patients with detrusor overactivity. Together, this can introduce new diagnostic, prognostic, and predictive microbiome-based biomarkers in clinical urology practice [19].

The most abundant bacteria found in the urinary microbiome in healthy women is *Lactobacillus* [20,21,22]. On the other hand, some *Lactobacillus* species have been suggested to be associated with pathological state, such as *L. gasseri* that have been associated with urgency incontinence, while *L. crispatus* may have a more protective effect. [7] This correlates with our findings that *L. gasseri* is reported more frequently in OAB-DO+ group compared to controls, while none of the samples from this group showed presence of *L. crispatus*. These published results are in line with our findings. We also found, *Lactobacillus* to be the dominant bacteria that was found in our study groups, followed by *Gardnerella* and *Prevotella*.

The median proportion of *Lactobacillus* in the OAB DO+ group was significantly higher compared to the OAB DO- and control group (Table 1). Looking on the species level, 58% of the samples from the OAB DO+ group showed a dominance of *Lactobacillus iners,* which was much higher compared to OAB-DO minus and controls (Table 2).

There are four studies that have shown a decrease in *Lactobacillus* in patients with OAB or urge incontinence [7,8,20,23]. The other studies did not include urodynamics and included patients with self-reported OAB symptoms [7,8,13,14]. Our results show that there were considerable differences in the microbiome between OAB subtypes that could explain differences in reported *Lactobacillus* presence in these other studies.

*Gardnerella* proportions did not differ significantly between our study groups. Previous studies have also shown *Gardnerella* to be equally present in their study groups [24]. Nonetheless, one study did show increased *Gardnerella* in OAB patients [7].

Median *Prevotella* proportions were significantly smaller in OAB DO+ than in OAB DO- and in control patients in our study. These findings are in agreement with previous reported studies, which have shown significantly less *Prevotellacea* in urgency urinary incontinence patients compared to controls [13,24].

Our results showed *Propionimicrobium, Dialister, Anaerococcus,* and *Peptoniphilus* species made up significantly smaller proportions of the microbiome in the OAB DO+ group compared to one or both of the other patient groups. These species were not significantly downregulated in other studies that reported genera, including *Proteus, Aerococcus, and Enterococcus*, to be increased and genera, such as *Staphylococcus*, to be decreased in patients with OAB compared to controls [9,25].

There are currently many studies demonstrating correlations between the microbiome and OAB presence and symptoms. Distinguishable microbiota have previously been identified between different forms of incontinence [11]. The microbiota of patients with pure stress incontinence was not investigated in our study, and therefore, no conclusions can be made regarding differentiation between OAB and other forms of urine incontinence based on our data. This study showed that OAB patients with proven detrusor overactivity have a significantly less diverse microbiota than OAB patients who do not have this characteristic. This is in line with earlier reports that an increase in the severity of OAB symptoms was associated with a reduction in microbial diversity [24].

Whether total bacterial load is of importance is still unclear, but there is a study that demonstrated that the presence of bacterial DNA in the urine is associated with an increased frequency of urgency urinary incontinence episodes [26,27]. One study reported an association between OAB and bacteria usually associated with UTIs, such as *E. coli* and *Proteus* [24]. Our cohort did not detect any significant changes in common UTI-associated bacteria, but two patients in our OAB-DO+ group did have a clear microbiome dominance of an Enterobacter species. The Enterobacteriaceae detected in our analyses are most likely Escherichia coli bacteria [28]. 

It is still unclear if microbiome correction can reduce OAB symptoms. One interesting study investigated this in OAB patients who were treated with anticholinergics. Contrary to our study, the authors reported an increased diversity in OAB patients versus controls. The treatment non-responders had significantly more bacterial load and decreased microbiome diversity compared to controls [29]. Also, lactobacillus-containing feminine hygiene products have been recently shown to have an effect on the vaginal microbiome and genitourinary symptoms in pre- and postmenopausal women and improve OAB symptoms [30]. Additionally, urinary microbiota in women may have an effect on the response to Botulinum neurotoxin-A treatment [23].

## 5. Conclusions

Significant differences were observed in the urinary microbiome of patients with overactive bladder syndrome who have detrusor overactivity on urodynamics compared to OAB patient without detrusor overactivity and matched controls. OAB patients with detrusor overactivity have a significantly less diverse microbiome and show a higher proportion of *Lactobacillus,* particularly *Lactobacillus iners.*

Results imply that the urinary microbiome could be involved in the pathogenesis of a specific phenotype of OAB. The urinary microbiome could be a new starting point to study the causes and treatments of OAB.

## Figures and Tables

**Table 1:** Differences in proportions of bacteria in the microbiota ISKW test was used to compare the median sequence abundances between the cohorts.

Family	OAB DO+ ^a^	OAB DO- ^a^	Control ^a^	ISKW-Test ^b^	Pairwise Comparisons of Groups ^b^		
					OAB DO+ /OAB DO-	OAB DO+ /Control	OAB DO- /Control
*Lactobacillus*	57.33 (74.60)	0.04 (21.44)	0.03 (17.82)	0.024 *	0.041 *	0.011 *	0.753
*Prevotella*	5.13 (8.82)	12.58 (17.35)	23.48 (17.42)	0.006 *	0.013 *	0.004 *	0.840
*Gardnerella*	0.27 (24.79)	0.01 (3.76)	12.94 (46.41)	0.356	X	x	X
*Mobiluncus*	0.01 (0.07)	0.23 (0.71)	0.01 (0.05)	0.064	X	x	X
*Corynebacterium*	0.04 (0.23)	0.11 (0.61)	0.00 (1.31)	0.907	X	x	X
*Propionimicrobium*	0.00 (0.06)	0.31 (1.45)	0.09 (0.33)	0.007 *	0.002 *	0.125	0.080
*Bifidobacterium*	0.00 (0.01)	0.00 (0.05)	0.00 (0.00)	0.795	X	x	X
*Atopobium*	0.00 (0.07)	0.00 (0.05)	0.00 (2.82)	0.785	X	x	X
*Bacteroides*	0.00 (0.00)	0.01 (0.07)	0.00 (0.07)	0.469	X	x	X
*Porphyromonas*	0.05 (0.15)	1.52 (9.52)	0.12 (4.45)	0.107	X	x	X
*Aerococcus*	0.00 (0.10)	0.07 (0.47)	0.00 (5.58)	0.409	X	x	X
*Facklamia*	0.00 (0.07)	0.01 (0.31)	0.00 (0.12)	0.495	X	x	X
*Streptococcus*	0.05 (0.40)	0.00 (0.69)	3.59 (6.80)	0.121	X	x	X
*Clostridialis*	0.00 (0.01)	0.05 (1.52)	0.00 (0.00)	0.041 *	0.028 *	0.930	0.022 *
*Dialister*	0.32 (0.86)	2.01 (5.12)	2.70 (1.46)	<0.001 *	0.003 *	<0.001 *	0.774
*Anaerococcus*	0.14 (0.89)	6.02 (9.01)	1.44 (6.33)	0.004 *	0.001 *	0.025 *	0.250
*Finegoldia*	0.31 (1.73)	0.46 (5.81)	2.81 (6.26)	0.264	X	x	X
*Peptoniphilus*	0.27 (1.27)	2.73 (2.77)	2.84 (3.43)	0.008 *	0.016 *	0.004 *	0.799
*Fusobacterium*	0.02 (0.07)	0.00 (0.73)	0.00 (0.59)	0.877	X	x	X
*Sneathia*	0.00 (0.00)	0.00 (20.79)	0.00 (0.00)	0.171	X	x	X
*Campylobacter*	0.32 (0.70)	1.34 (2.56)	0.59 (3.59)	0.271	X	x	X
*Enterobacteriacae*	0.06 (0.95)	0.00 (0.15)	0.23 (2.98)	0.131	X	x	X
*Haemophilus*	0.00 (0.00)	0.00 (0.00)	0.00 (0.00)	0.761	X	x	X
*Ureaplasma*	0.00 (0.49)	0.00 (0.00)	0.00 (0.00)	0.272	X	x	X

ISKW test: Independent Sample Kruskal–Wallis Test. ^a^ presented as median (interquartile range). ^b^ presented as significance, * marks significance a of *p* < 0.05.

**Table 2:** Dominance of *Lactobacillus iners*.

Dominant Lactobacillus	OAB DO+ ^a^ (n = 12)	OAB DO- ^a^ (n = 9)	Control ^a^ (n = 12)	Total (n = 33)
*L. iners*	58% (52.9)	22% (27.6)	8% (42.4)	30%
*L. gasseri*	25% (8.3)	0% (-)	17% (27.3)	15%
*L. crispatus*	0% (-)	11% (16.1)	0% (-)	3%

^a^ presented as percentage of patients (median proportion).

## Data Availability

Raw data can be requested from the corresponding author.
